# Implementation of Ambipolar Polysilicon Thin-Film Transistors with Nickel Silicide Schottky Junctions by Low-Thermal-Budget Microwave Annealing

**DOI:** 10.3390/nano12040628

**Published:** 2022-02-13

**Authors:** Jin-Gi Min, Dong-Hee Lee, Yeong-Ung Kim, Won-Ju Cho

**Affiliations:** Department of Electronic Materials Engineering, Kwangwoon University, Seoul 01897, Korea; wlsrl1659@naver.com (J.-G.M.); zpsxlzje@naver.com (D.-H.L.); jobs0506@naver.com (Y.-U.K.)

**Keywords:** thin-film transistors, microwave annealing, rapid thermal annealing, ambipolar conduction characteristics, silicide, Schottky junctions

## Abstract

In this study, the efficient fabrication of nickel silicide (NiSi*_x_*) Schottky barrier thin-film transistors (SB-TFTs) via microwave annealing (MWA) technology is proposed, and complementary metal-oxide-semiconductor (CMOS) inverters are implemented in a simplified process using ambipolar transistor properties. To validate the efficacy of the NiSi*_x_* formation process by MWA, NiSi*_x_* is also prepared via the conventional rapid thermal annealing (RTA) process. The Rs of the MWA NiSi*_x_* decreases with increasing microwave power, and becomes saturated at 600 W, thus showing lower resistance than the 500 °C RTA NiSi*_x_*. Further, SB-diodes formed on n-type and p-type bulk silicon are found to have optimal rectification characteristics at 600 W microwave power, and exhibit superior characteristics to the RTA SB-diodes. Evaluation of the electrical properties of NiSi*_x_* SB-TFTs on excimer-laser-annealed (ELA) poly-Si substrates indicates that the MWA NiSi*_x_* junction exhibits better ambipolar operation and transistor performance, along with improved stability. Furthermore, CMOS inverters, constructed using the ambipolar SB-TFTs, exhibit better voltage transfer characteristics, voltage gains, and dynamic inverting behavior by incorporating the MWA NiSi*_x_* source-and-drain (S/D) junctions. Therefore, MWA is an effective process for silicide formation, and ambipolar SB-TFTs using MWA NiSi*_x_* junctions provide a promising future for CMOS technology.

## 1. Introduction

Metal silicides, i.e., compounds of metal and silicon (Si), have been widely employed as interconnecting and contact materials in complementary metal-oxide-semiconductor (CMOS) technology due to their low specific resistivity, low contact resistivity towards both types of Si, high thermal stability, good processibility, and excellent process compatibility with standard Si technology [1,2,3,4]. Transition-metal silicide-based Schottky barrier (SB) source-and-drain (S/D) junctions have aroused much interest in nanoscale metal-oxide-semiconductor field-effect transistors (MOSFETs) because the resistive–capacitive (RC) time delay must be reduced by minimizing both parasitic resistance and capacitance components, in order to meet the major requirement of speeding up electronic circuits [5,6,7]. In SB-MOSFETs, the S/D junction consists of silicide in place of conventional impurity-doped silicon, thus enabling lower parasitic series resistance and ultrashallow abrupt junction formation via a simpler process [7,8,9,10]. By contrast, the performance of conventional MOSFETs is determined by doping and activation techniques, which become increasingly difficult for ultra-short-channel devices. Nevertheless, the Ti silicides (TiSi*_x_*) and Co silicides (CoSi*_x_*) that are commonly used in CMOS fabrication have limitations in future, extremely scaled down, ultra-high-density CMOS electronic circuits. In the case of TiSi*_x_*, the sheet resistance (R_s_) increases as the line width decreases [11,12,13,14], while in the case of CoSi*_x_*, junction spiking becomes an issue due to excessive Si consumption [12,15,16]. Meanwhile, Ni silicide (NiSi*_x_*) is gaining attention in next-generation deep submicron CMOS devices due to its improved nanoscale performance, and is gradually replacing CoSi*_x_* [17]. In particular, there are many advantages, such as low specific resistivity (10–15 μΩ cm) and formation temperature (typically 500 °C), decreased Si consumption (1.83 nm of Si per nanometer of Ni, yielding 2.21 nm of NiSi*_x_*), and little deterioration in resistivity on narrow lines/gates [16,18,19,20,21,22]. In particular, NiSi*_x_* materials are the standard metal contacts in the semiconductor industry for both NMOS and PMOS devices, and are regarded as midgap metals with Schottky barrier heights (SBHs) of 0.45–0.5 eV for holes, and 0.6–0.65 eV for electrons [23,24]. Therefore, when applied to the S/D metallic junctions for Schottky barrier thin-film transistors (SB-TFTs), NiSi*_x_* is particularly favorable for obtaining ambipolar operating characteristics without n-type or p-type impurity doping. Consequently, these devices are able to behave as p-type or n-type MOSFETs simply by changing the polarity of the gate bias.

The traditional silicide formation process has generally involved conventional rapid thermal annealing (RTA) using a halogen lamp. However, because the process is typically performed in a vacuum, RTA has the disadvantages of high cost, relatively long processing time, and high thermal budget [25,26]. By contrast, microwave annealing (MWA) does not require a vacuum, is cheaper, and has higher energy-transfer efficiency and consumption, a shorter process time, and a lower thermal budget [27,28]. There are several studies of applying MWA with these advantages to the activation of ion-implanted dopants [29,30]. In addition, MWA can offer quicker volume heating than the RTA, because it interacts directly with individual atoms while inducing dipole rotation in the silicon substrates [31]. Therefore, MWA is employed herein to promote the silicide reaction between Ni and Si. To verify the efficiency of silicidation by MWA, conventional RTA is also applied to NiSi*_x_* formation for comparison. The crystallinity and R_s_ values of the NiSi*_x_* prepared via MWA at various microwave powers, and by RTA at 500 °C, are evaluated. In addition, NiSi*_x_* SB-diodes are fabricated via MWA at various microwave powers, and their electrical characteristics are measured to determine the optimum fabrication conditions. These conditions are then used to fabricate ambipolar NiSi*_x_* SB-TFTs, and their electrical characteristics and reliability are evaluated in comparison with identical devices prepared via RTA. Further, CMOS-like inverters are constructed using the MWA- or RTA-NiSi*_x_* SB-TFTs, in order to evaluate their voltage transfer characteristics, voltage gains, and dynamic inversion operation.

## 2. Materials and Methods

### 2.1. Nickel Deposition and Silicidation

The substrates were (100)-oriented n-type and p-type Si wafers with resistivities ranging from 10 to 20 Ω·cm. The substrates were cleaned using the process recommended by the Standard Radio Corporation of America (RCA) to remove any surface contamination and native oxides. Active regions were then formed via a photolithographic patterning process and wet etching with a 30:1 buffered oxide etchant (BOE). A 150 nm-thick Ni film was deposited using an electron-beam (E-beam) evaporator. After that, the MWA process was performed at powers of 250–1000 W under a N_2_ atmosphere for 2 min for NiSi*_x_* formation. For comparison, the RTA process was conducted at 500 °C for 2 min under a N_2_ atmosphere. After that, the unreacted Ni was removed using a 1:1 sulfuric acid/hydrogen peroxide mixture (SPM) at room temperature. 

**Figure 1:**
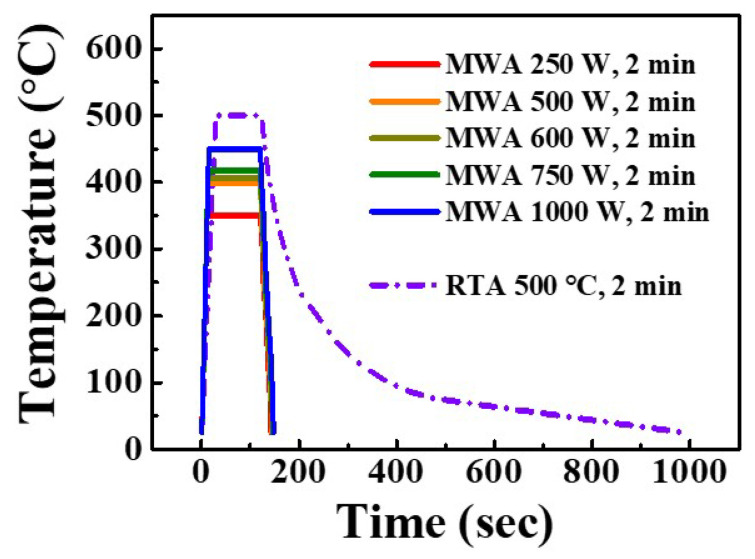
Temperature profiles for the MWA process at 250–1000 W, and the RTA process at 500 °C.

The temperature profiles of the MWA and RTA processes are shown in Figure 1. We used an infrared (IR) thermometer to check the temperature during the MW treatment process, because it is hard to determine the temperature inside the MW chamber using metal thermocouples [32]. Thus, after heat treatment at 500 °C via the RTA process, about 15 min are required to return to room temperature, and the thermal budget is 2.84 × 10^5^ °C·s. By contrast, the MWA process is a volumetric heating method using electromagnetic waves, which reaches the process temperature within about 20 s and has a very short ramp downtime of 10 s. The thermal budget of the MWA process is 0.47 × 10^5^, 0.54 × 10^5^, 0.56 × 10^5^, 0.59 × 10^5^, and 0.65 × 10^5^ °C·s at operating powers of 250, 500, 600, 750, and 1000 W, respectively. This indicates that the MWA process generally has a lower thermal budget and a higher energy transfer efficiency than the RTA process. 

### 2.2. Fabrication of the NiSi_x_ SB-Diodes 

**Figure 2:**
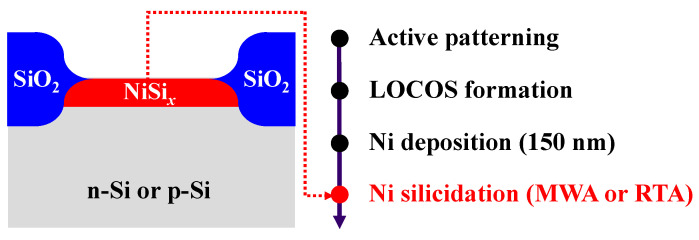
The schematic structure and process flow of the NiSi*_x_* SB-diodes.

As shown schematically in Figure 2, phosphorus- and boron-doped (100) n-type and p-type bulk silicon wafers with resistivities of 3–5 and 7–14 Ω∙cm, respectively, were used to fabricate the NiSi*_x_*-based SB-diodes. After defining the active area of the diode, a 500 nm-thick SiO_2_ layer was grown via wet oxidation at 980 °C for local oxidation of silicon (LOCOS) isolation. A 150 nm-thick Ni film was deposited using an E-beam evaporator. For NiSi*_x_* formation, either the MWA process at a power of 250–1000 W for 2 min, or the RTA process at 500 °C for 2 min, was employed under a N_2_ atmosphere, to investigate the effect of MWA silicidation upon the characteristics of the SB-diodes. Then, unreacted Ni was removed using an SPM solution at room temperature. 

### 2.3. Fabrication of the NiSi_x_ SB-TFTs 

**Figure 3:**
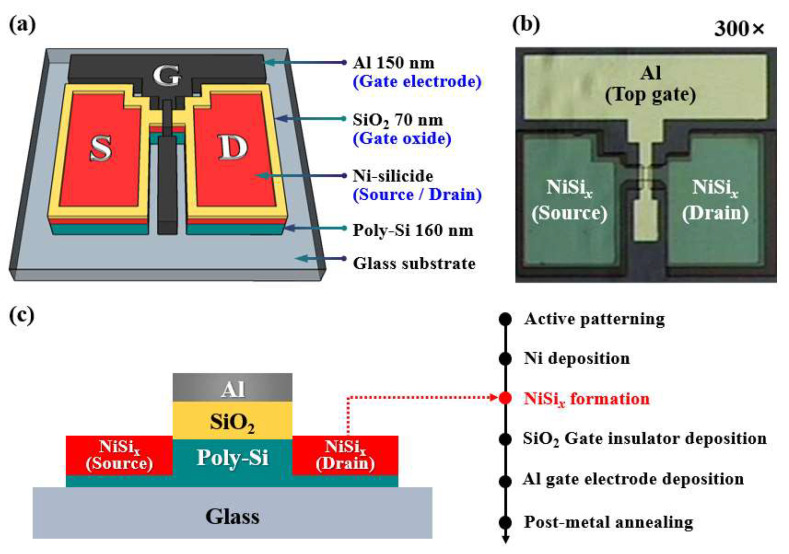
The schematic structure (**a**), top-view optical microscope image (**b**), and cross-sectional structure and process flow (**c**) of the fabricated NiSi*_x_* SB-TFTs.

Glass substrates (1737 and EAGLE2000TM, Corning) were each coated with a 160 nm-thick poly-Si layer via excimer-laser annealing (ELA) for use in fabricating the SB-TFTs with NiSi*_x_* S/D junctions. The resulting ELA poly-Si substrate was then cleaned via the RCA process, after which the active channel regions were patterned via photolithography and wet etching with a 30:1 BOE solution. The channel width (W) and length (L) of the fabricated devices were 20 µm and 10 µm, respectively. Immediately after removing the native oxide film from the poly-Si channel with the 30:1 BOE, a 150 nm-thick Ni film was deposited using an E-beam evaporator. Subsequently, NiSi*_x_* was selectively formed in the S/D region via the MWA process at 600 W, which is the optimal power condition. Then, unreacted Ni was removed using the SPM solution. For the gate insulator, a 70 nm-thick SiO_2_ film was deposited by radio-frequency (RF) magnetron sputtering at an operating pressure of 3.0 mTorr, an Ar flow rate of 30 sccm, an O_2_ flow rate of 2 sccm, and an RF power of 200 W. For the top-gate electrode, a 150 nm-thick Al film was deposited using an E-beam evaporator, then patterned by a lift-off method. Finally, post-metallization annealing (PMA) was performed using forming gas (5% H_2_, 95% N_2_) in a furnace at 400 °C for 30 min to improve the electrical properties. The schematic structure, process flow, and top-view optical microscope image (300×) of the fabricated NiSi*_x_* SB-TFTs are presented in Figure 3. 

### 2.4. Characterization of the NiSi_x_ SB-Diodes and SB-TFTs 

The electrical characteristics of the fabricated NiSi*_x_* Schottky junction diodes and the SB-TFTs were measured using an Agilent 4156B precision semiconductor parameter analyzer in a dark box to prevent external effects such as light and electrical noise. In addition, CMOS inverters were constructed using the MWA- and RTA-processed NiSi*_x_* SB-TFTs, and their voltage transfer characteristics, voltage gains, and dynamic inversion behaviors were evaluated using an RIGOL DG972 function/arbitrary waveform generator and RIGOL MSO5074 oscilloscope in a dark box.

## 3. Results and Discussion

**Figure 4:**
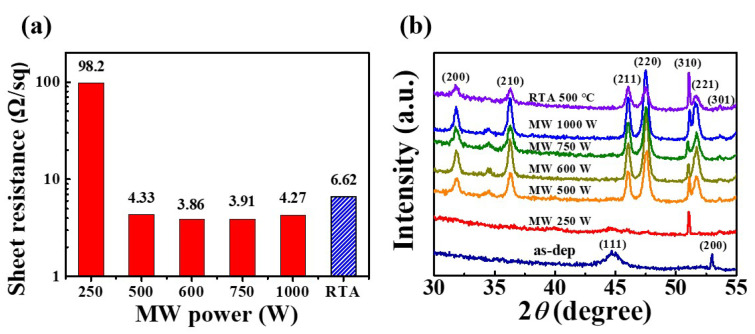
The R_s_ values (**a**) and XRD spectra (**b**) of the NiSi*_x_* samples obtained via MWA at powers of 250–1000 W for 2 min, or via RTA at 500 °C for 2 min, under a N_2_ atmosphere.

The sheet resistances (R_s_) of the NiSi*_x_* samples fabricated at various microwave powers were measured using a four-point probe, and the results are presented in Figure 4a. Here, a significant decrease in R_s_ is observed at 500 W, thus resulting in low resistance. In particular, the silicidation process at 600 W provides the lowest R_s_ value of 3.86 Ω/sq, which is lower than the 6.62 Ω/sq obtained via the RTA process at 500 °C. These results are similar to those reported in other literature [33]. Therefore, it is concluded that efficient silicide formation is possible even with a low-power MWA process, by sufficiently reducing the resistance. 

The crystallinities of the various NiSi*_x_* samples are indicated by the XRD spectra in Figure 4b. Here, the XRD pattern of the as-deposited Ni film exhibits peaks corresponding to the (111) and (200) crystal planes of Ni, while various other peaks appear after the RTA and MWA silicidation processes. In particular, the MWA treatment leads to the appearance of a peak corresponding to the (310) crystal plane of NiSi*_x_*, even at a low microwave power of 250 W, thus confirming the formation of silicide. When the MWA process is performed at 500 W, several strong peaks corresponding to the (211), (220), (310), (221), and (301) crystal planes also appear [34], giving almost the same pattern as that obtained using the 500 °C RTA process. These results indicate that 600 W is the optimal MWA silicidation condition.

**Figure 5:**
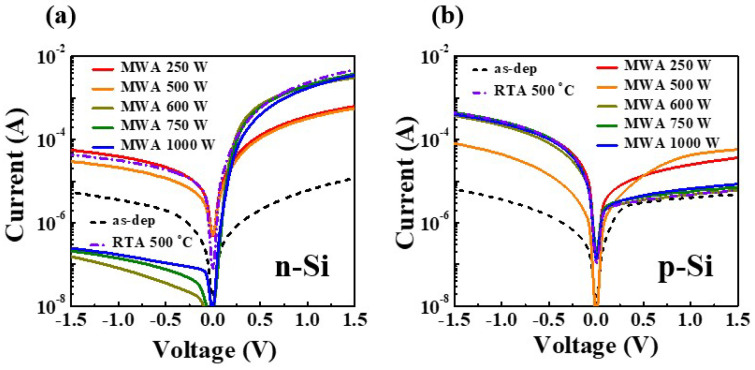
The current–voltage (I-V) curves of the NiSi*_x_* SB-diodes on (**a**) n-type and (**b**) p-type Si substrates according to the various silicidation conditions.

The current–voltage (I-V) characteristics of the NiSi*_x_* Schottky junction diodes on n-type and p-type Si substrates according to the various silicidation conditions are presented in Figure 5a and Figure 5b, respectively, and the corresponding electrical parameters are summarized in Table 1. The as-deposited Ni SB-diode has a low on-current and a high leakage current due to interfacial defects between the unreacted Ni film and Si. However, as silicidation proceeds, the rectification characteristics of the n-type and p-type diodes are improved, and the on/off current ratio increases. In particular, the MWA process improves the operating performance as the microwave power increases, and exhibits the best rectification characteristics at 600 W. Notably, the 600-W MWA SB-diodes on the n-type substrate provide better results than the 500 °C RTA diodes. This is also evidenced by the ideality factors (*η*) extracted from the I-V curves, and the Schottky barrier heights (ϕ_b_) extracted from the current–voltage (I-V) curves (Table 1); moreover, it is consistent with the Rs and XRD results. In addition, the ϕ_b_ values were extracted by Equation (1) [35]:

(1) $$\phi_{b}(V)=\phi_{b0}+\left(\frac{n-1}{n}\right)V$$

where V, *n* are voltage and ideality factor, respectively.

**Figure 6:**
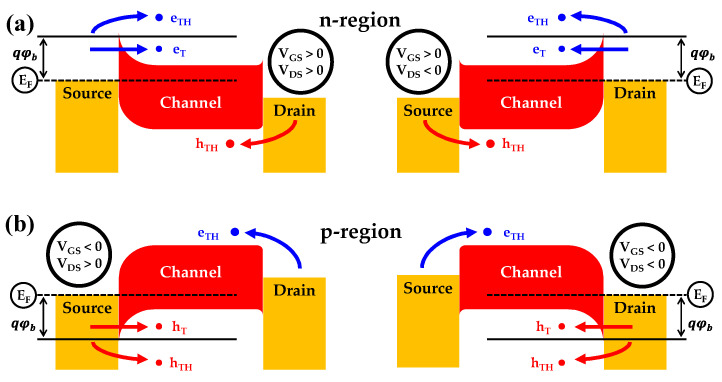
Band diagram of MWA- and RTA-treated ambipolar NiSi*_x_* SB-TFTs in schematic form based on VGS and VDS (**a**) n-region operation (**b**) p-region operation.

Figure 6 shows schematic energy band diagrams based on V_GS_ and V_DS_ values for ambipolar operation. The current device is mainly related to thermionic emission and tunneling of carriers above the threshold in the SB-TFTs. The electrons are injected into the channel using thermionic emission (e_TH_) and tunneling (e_T_) from the source-and-drain electrodes at the positive and negative V_DS_, when the gate bias is positive (n-channel operation), as shown in Figure 6a. In Figure 6b, the holes are injected into the channel region by thermionic emission (h_TH_) and tunneling (h_T_) at the negative gate bias (p-channel operation). Depending on the operating mode, electrodes or holes from source-and-drain electrodes fill the channel region during the ambipolar operation. As a result, the driving current and I_on_/I_off_ of p-channel operation can be lower, because the SB height (SBH) for electrons in NiSi*_x_* is slightly higher than the SBH for holes, but the effective electron mass of tunneling is lower.

**Figure 7:**
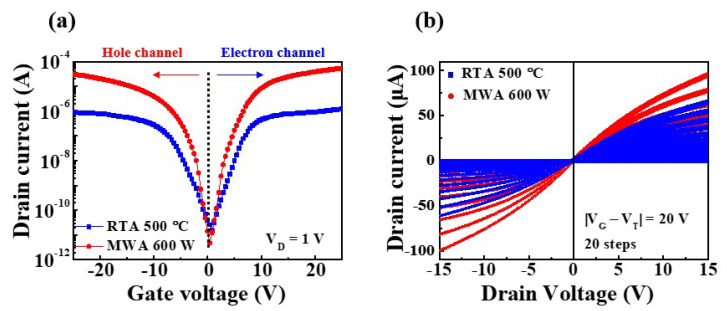
The transfer curves (**a**) and output curves (**b**) of the 600-W MWA and 500-°C RTA NiSi*_x_* SB-TFTs.

The electrical properties of the NiSi*_x_* SB-TFTs fabricated via the MWA silicidation process at 600 W, and via the RTA process at 500 °C, are presented in Figure 7. The transfer curves measured at a drain voltage (*V_D_*) of 1 V and a gate voltage (*V_G_*) range of −25 to +25 V (Figure 7a) demonstrate the ambipolar conduction properties of both devices. Meanwhile, the output curves measured at |*V_G_* − *V_TH_*| = 0–20 V (where *V_TH_* is the threshold voltage) in the drain voltage range of −15 to +15 V demonstrate that both devices can behave as p-type (hole channel) or n-type (electron channel) MOSFETs simply by changing the polarity of the gate bias. The drain current (*I_D_*) increases linearly in the low *V_D_* region, indicating a pinch-off characteristic that gradually reaches the saturation region as *V_D_* increases further. Taken together, these results indicate that the 600-W MWA NiSi*_x_* SB-TFT allows better switching characteristics and higher drive current than the 500-°C RTA-processed device. This is also consistent with the results obtained for the SB-diode. 

The extracted electrical parameters of the 600-W MWA and the 500-°C RTA NiSi*_x_* SB-TFTs are summarized in Table 2. The subthreshold swing (*SS*) and field-effect mobility (*μ*_*FE*_) values were extracted using Equations (2) and (3) [36]:

(2) $$SS={\left[\left(\frac{dlogI_{D}}{dV_{G}}\right)\right]}^{-1}$$

and

(3) $$\mu_{FE}=\left(\frac{Lg_{m}}{W\cdot C_{ox}\cdot V_{D}},\;g_{m}=\frac{\partial I_{D}}{\partial V_{G}}\right)$$

where *L*, *W*, *g_m_*, and *C_ox_* are the channel length, width, transconductance, and gate oxide capacitance per unit area, respectively. Thus, the 600-W MWA NiSi*_x_* SB-TFT exhibits an *SS* of 633.4 mV/dec, a *μ*_*FE*_ of 16.5 cm^2^/V·s, and a *V_TH_* of 2.3 V during p-type behavior, and an *SS* of 629.2 mV/dec, a *μ*_*FE*_ of 20.3 cm^2^/V·s, and a *V_TH_* of −1.4V during n-type behavior. Meanwhile, the 500-°C RTA NiSi*_x_* SB-TFT exhibits an *SS* of 1201.1 mV/dec, a *μ*_*FE*_ of 4.9 cm^2^/V·s, and a *V_TH_* of 3.4 V during p-type behavior, and an *SS* of 1321.4 mV/dec, a *μ*_*FE*_ of 4.1 cm^2^/V·s, and a *V_TH_* of −2.4 V during n-type behavior. Thus, the NiSi*_x_* SB-TFTs fabricated by RTA at 500 °C have a higher leakage current and poorer electrical parameters than those obtained by MWA at 600 W. Meanwhile, the *SS* and high *μ*_*FE*_ determine the power consumption and switching performance.

**Figure 8:**
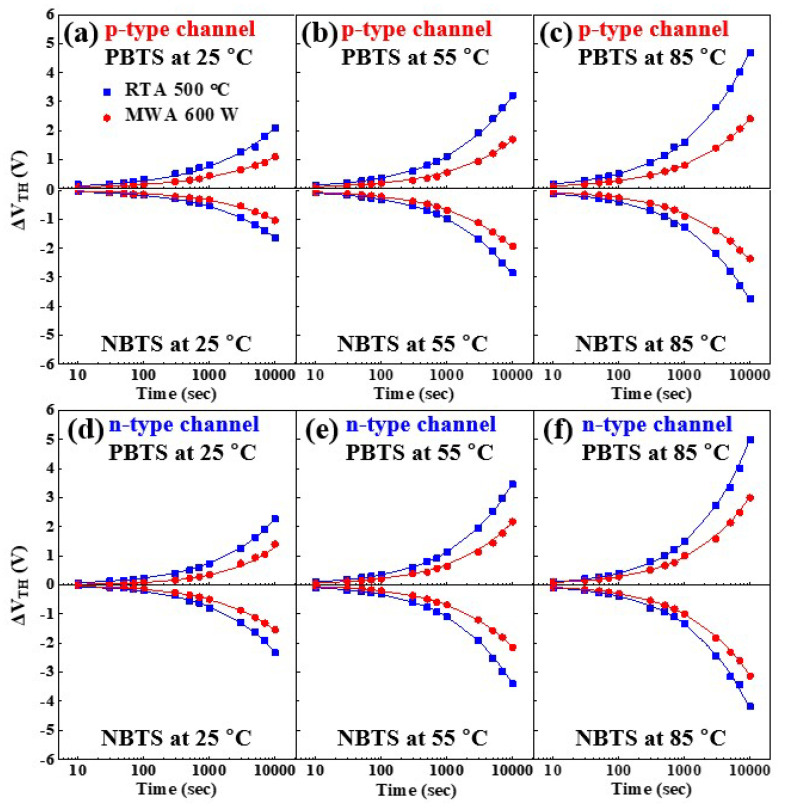
The temperature dependence of Δ*V_TH_* in the p-type channel (**a**–**c**) and the n-type channel (**d**–**f**) of the MWA and RTA NiSi*_x_* SB-TFTs during the PBTS (V_G_ = +20 V) and NBTS (V_G_ = −20 V) tests for 10^4^ s: (**a**,**d**) 25 °C, (**b**,**e**) 55 °C and (**c**,**f**) 85 °C.

The temperature-dependence of the *V_TH_* shift (Δ*V_TH_*) during the positive bias temperature stress (PBTS) and negative bias temperature stress (NBTS) tests are indicated for the 600-W MWA and 500-°C RTA NiSi*_x_* SB-TFTs in Figure 8. The p-channel behavior is presented in Figure 8a–c, while the n-channel behavior characteristics are shown in Figure 8d–f. For these measurements, the change in *V_TH_* was monitored at 25, 55, and 85 °C while applying an electric field of ±20 V to the gate electrode for 10^4^ s. Due to the ambipolar nature of the NiSi*_x_* SB-TFTs, the p-channel and n-channel behaviors were tested separately. The fitted curves in Figure 8a–f were obtained using Equation (4) [37,38]:

(4) $$\Delta V_{TH}\left(t\right)=\Delta V_{TH0}\left\{1-\exp\left[-{\left(\frac{t}{\tau }\right)}^{\beta }\right]\right\}$$

where Δ*V_TH0_* is Δ*V_TH_* at the initial time, *β* is the exponent for a stretched-exponential function, and *τ* is the carrier trapping time from the channel to the dielectric layer, which depends on the temperature. Therefore, *V_TH_* and *τ* are dependent on the thermally activated process. The temperature-dependent effective energy barrier height (*E_τ_*) for carrier transport was calculated using the Arrhenius equation, given as Equation (5):

(5) $$\tau =\tau_{0}\exp\left(\frac{E_{\tau }}{k_{B}T}\right)=\nu^{-1}\exp\left(\frac{E_{\tau }}{k_{B}T}\right)$$

where *ν* and *τ*_0_ are, respectively, the frequency and the thermal pre-factor for emission over the barrier, and T is the absolute temperature. The results indicate that the Δ*V*_TH_ increases with increasing stress time, and with increasing stress temperature, in both behavior modes. Moreover, as the Δ*V*_TH_ of the MWA SB-TFT is smaller than that of the RTA device, MWA silicidation is considered to contribute to the improvement in stability and reliability of the SB-TFT. 

**Figure 9:**
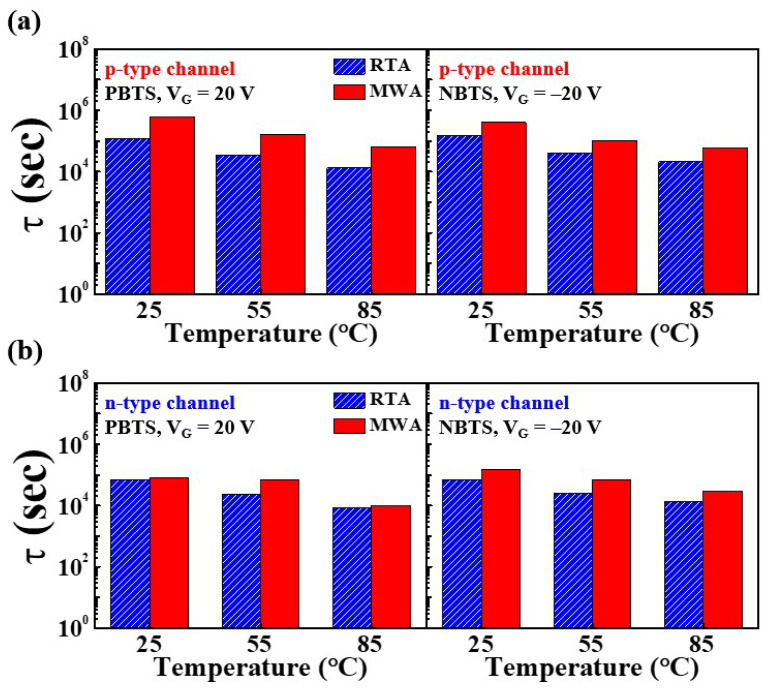
Box plots of the charge trapping time (*τ*) in the p-channel (**a**) and n-channel (**b**) behavioral modes of the MWA- and RTA-NiSi*_x_* SB-TFTs during the PBTS and NBTS tests.

The time it takes for the carrier to be trapped in an insulating layer or an insulating layer–channel layer is referred to as the charging trapping time (*τ*). The *τ* of the NiSi*_x_* SB-TFTs in the p-channel and n-channel behavioral modes during the PBTS and NBTS tests are plotted in Figure 9, and the extracted values are summarized in Table 3. In both modes, the trapping time is seen to decrease with increasing bias-stress-temperature. Moreover, the extracted results show that the charge trapping time in the PBTS mode is shorter than that in the NBTS mode, thus indicating that the PBTS is more dominant in charge trapping. Further, the trapping times of the MWA device are greater than those of the RTA device, thereby indicating that the MWA device is less susceptible to charge trapping in the PBTS and NBTS tests than is the RTA device.

**Figure 10:**
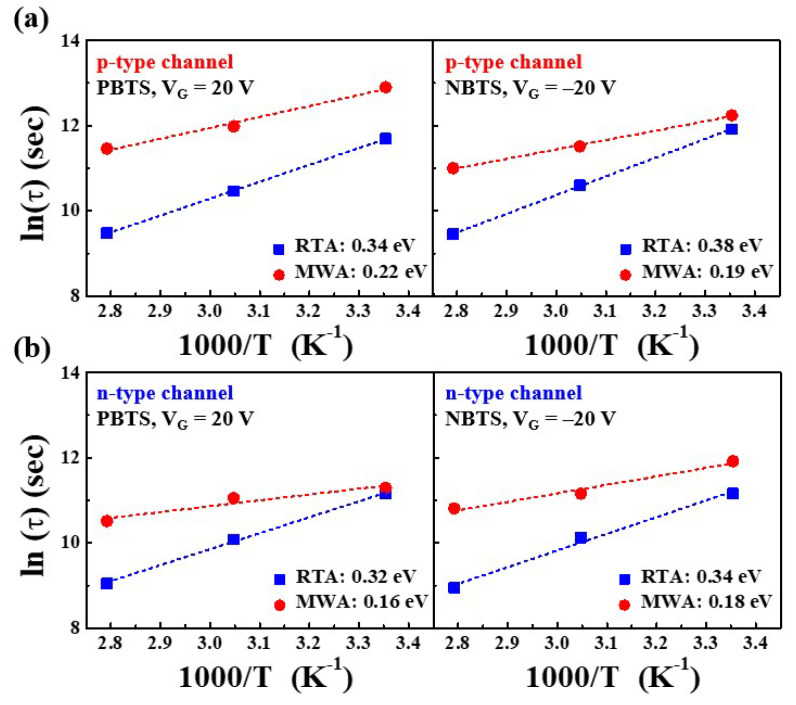
The charge trapping time (*τ*) of the MWA- and RTA-NiSi*_x_* SB-TFTs during the PBTS and NBTS tests as a function of temperature for the p-channel (**a**), and n-channel (**b**) modes.

Using the Arrhenius relationship, the logarithm of *τ* is plotted as a function of the inverse temperature for the p- and n-channels in Figure 10. These results suggest that the charge trapping process is driven by thermal activation. Hence, the trapping process of thermally activated charges is given by a linear relationship in ln(*τ*) versus 1/T. Thus, from Equation (5), the slope of the Arrhenius plot in the PBTS and NBTS tests represents the average effective barrier height (*E_τ_*) for charge transport. Estimates of *E_τ_* for the MWA and RTA NiSi*_x_* SB-TFTs during the PBTS and NBTS tests are summarized in Table 4. Lower *E_τ_* has been found in several previous studies due to the more organized structure of the channel *a*-IGZO [39]. As *E_τ_* is smaller in MWA NiSi*_x_* SB-TFTs than in RTA NiSi*_x_* SB-TFTs, MWA processing leads to a more ordered *a*-IGZO structure than the RTA method.

**Figure 11:**
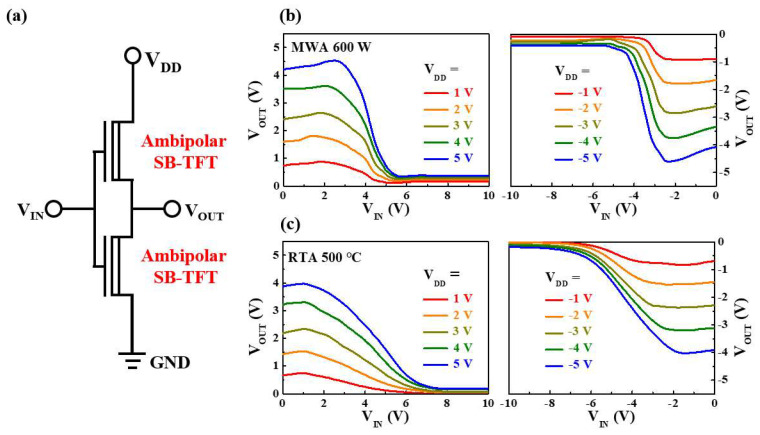
(**a**) Schematic diagram of an inverter circuit composed of two ambipolar NiSi*_x_* SB-TFTs; (**b**,**c**) the VTCs of the CMOS-like inverter in the first (left) and third (right) quadrants in (**b**) the MWA SB-TFT configuration (supply voltages V_DD_ = ± 1–5 V), and (**c**) the RTA SB-TFT configuration.

Finally, to validate the utility of the ambipolar NiSi*_x_* SB-TFTs, the operation of two types of CMOS-like inverter circuits is demonstrated according to the silicidation scheme. Two NiSi*_x_* SB-TFTs with identical geometry and channel dimensions were connected in order to construct a single inverter, as shown in the equivalent circuit in Figure 11a. The voltage transfer characteristics (VTCs) of the MWA and RTA devices at various supply voltages (V_DD_) are shown in Figure 11b,c, respectively. Here, typical and comparable ambipolar inverter behaviors are shown in the first (positive V_DD_ and V_IN_) and third (negative V_DD_ and V_IN_) quadrants of the inversion function. This is caused by the exchange of n- and p-channel behavior between the two TFTs, which represents the unique feature of the CMOS-like inverters constructed using the ambipolar NiSi*_x_* SB-TFTs. Thus, the SB-TFT connected to the V_DD_ side functions as a pull-up transistor, while the SB-TFT on the ground side operates as a pull-down transistor. 

**Figure 12:**
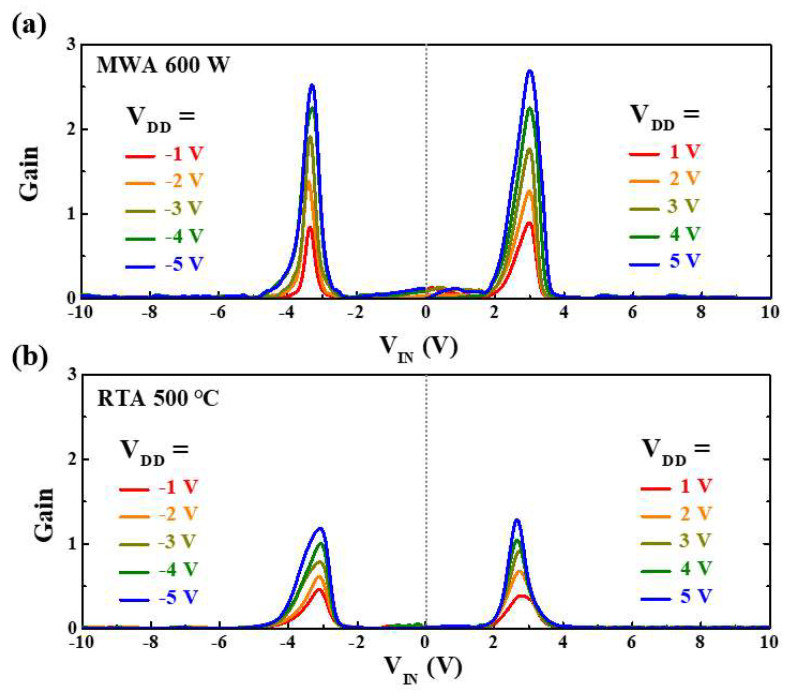
The voltage gains of the CMOS-like inverters in the first (right) and third (left) quadrants as a function of V_DD_: (**a**) the ambipolar MWA NiSi*_x_* SB-TFTs inverter, and (**b**) the ambipolar RTA NiSi*_x_* SB-TFTs inverter.

The extracted voltage gains (|∂V_OUT_/∂V_IN_|) in the first and third quadrants of the VTC curves at various V_DD_ values are plotted in Figure 12. In both inverter circuits, the voltage gain is seen to increase with increasing V_DD_. Moreover, the voltage gain is seen to be larger for the MWA device than for the RTA device, which is due to the high drive current and low leakage current of the MWA SB-TFTs. Accordingly, the MWA device exhibits superior inverter characteristics, and a steeper switching slope, than the RTA device. 

**Figure 13:**
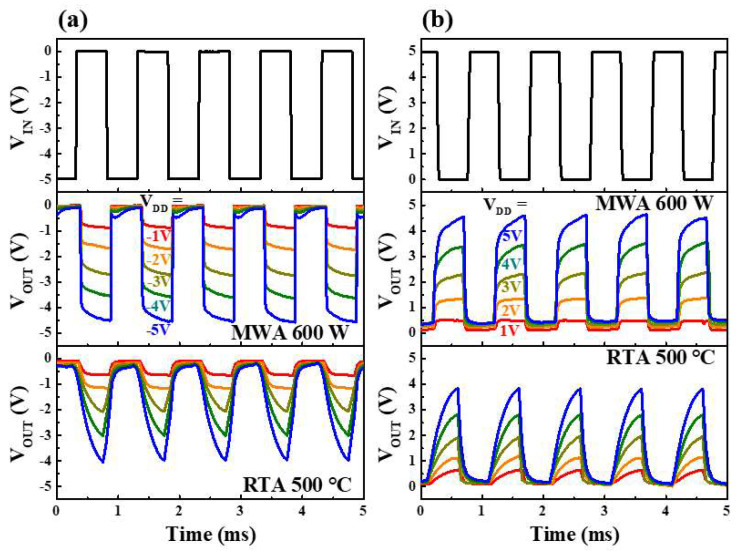
The dynamic inversion characteristics of the CMOS-like inverters in (**a**) the third and (**b**) the first quadrants as a function of V_DD_.

For circuit applications, it is necessary to understand the dynamic characteristics of the inverter. The dynamic inverting characteristics of the CMOS-like inverters are presented in Figure 13 for square-wave input signals at 1 KHz with various |V_DD_| values ranging from 1 V to 5 V. Figure 13a,b show the frequency response characteristics in the third and first quadrants, respectively. Here, the output voltage (V_OUT_) of the inverter is seen to increase with increasing V_DD_, and the MWA inverter displays an output waveform that is closer to the input signal (V_IN_) than does the RTA device. In addition, the MWA inverter can remain high and low for almost 0.5 ms, while the RTA inverter can only maintain low and difficult-to-maintain high-state operation. The MWA inverter has a high-state value of 4.53 V, and the RTA inverter has 4.04 V, which not only maintains a high state but also has better V_OUT_ characteristics of the high state. This is the result of the *SS* and *μ*_*FE*_ characteristics shown in Table 2. Based on excellent electrical properties, the MWA-NiSi*_x_* SB-TFTs exhibit superior high-speed response capability than the RTA-NiSi*_x_* SB-TFTs, which works the same when configuring inverter circuits. Therefore, Ni silicidation via MWA improves the operating characteristics of the SB-TFT, thereby enabling improved performance of complementary logic gates and faster frequency response. 

## 4. Conclusions

Herein, high-performance ambipolar nickel silicide (NiSi*_x_*) Schottky barrier thin-film transistors (SB-TFTs) were fabricated on excimer-laser-annealed (ELA) poly-Si substrates via a microwave annealing (MWA) process. For comparison, the conventional rapid thermal annealing (RTA) process was also utilized for the formation of NiSi*_x_*. The MWA process was shown to provide advantages such as higher energy transfer efficiency, along with a lower power consumption and thermal budget, than the RTA process. In addition, MWA is effective as a selective heating process, especially for thin-metal films. Prior to manufacturing the NiSi*_x_* SB-TFTs, SB-diodes were fabricated on bulk-Si substrates in order to evaluate the crystallinity and sheet resistance (R_s_) of the NiSi*_x_* prepared using MWA and RTA. The R_s_ of the MWA NiSi*_x_* was shown to decrease with increasing MW power, with the lowest R_s_ of all (3.86 Ω/sq) being obtained at 600 W. The MWA SB-diodes on n-type and p-type bulk-Si substrates showed better rectification operation and electrical characteristics than the RTA SB-diodes. In addition, NiSi*_x_* SB-TFTs were fabricated on ELA poly-Si substrates under identical conditions, and their electrical properties were compared. The results indicated that the MWA NiSi*_x_* SB-TFTs exhibit better electrical characteristics than the RTA devices, including the subthreshold swing (*SS*), field-effect mobility, threshold voltage (*V_TH_*), on/off current ratio (I_on_/I_off_) and interface state density (D_it_). Furthermore, the MWA NiSi*_x_* SB-TFTs exhibited lower threshold voltage shifts during the PBTS and NBTS tests, along with enhanced stability. In addition, complementary metal-oxide-semiconductor (CMOS) inverters with better VTC, gains, and excellent dynamic inversion characteristics in both the first and third quadrants were successfully constructed using the ambipolar MWA SB-TFT NiSi*_x_* S/D junctions. Therefore, ambipolar SB-TFTs containing NiSi*_x_* junctions prepared via the MWA process provide a prospective CMOS technology, because MWA is an excellent method for silicidation due to its high energy transfer efficiency, low power consumption, low thermal budget, and selective heating capacity.

## Figures and Tables

**Table 1:** The electrical parameters of n-type and p-type NiSi*_x_* SB-diodes obtained by various silicide processes.

Parameters	Type	As−dep	MWA					RTA
			250 W	500 W	600 W	750 W	1000 W	500 °C
**On current [A]**	**n**	1.2 × 10^−5^	6.3 × 10^−4^	5.7 × 10^−4^	3.1 × 10^−3^	3.8 × 10^−3^	3.5 × 10^−3^	4.8 × 10^−3^
	**p**	6.4 × 10^−6^	4.1 × 10^−4^	8.1 × 10^−5^	3.8 × 10^−4^	4.5 × 10^−4^	4.1 × 10^−4^	4.5 × 10^−4^
**Off current [A]**	**n**	5.4 × 10^−6^	5.6 × 10^−5^	3.0 × 10^−5^	1.6 × 10^−7^	2.1 × 10^−7^	2.5 × 10^−7^	4.3 × 10^−5^
	**p**	4.8 × 10^−6^	3.7 × 10^−5^	5.8 × 10^−5^	6.0 × 10^−6^	7.2 × 10^−6^	8.6 × 10^−6^	6.0 × 10^−6^
**On/Off ratio**	**n**	2.2	1.1 × 10^1^	1.9 × 10^1^	2.0 × 10^4^	1.8 × 10^4^	1.4 × 10^4^	1.1 × 10^2^
	**p**	1.4	1.1 × 10^1^	1.4	6.3 × 10^1^	6.2 × 10^1^	4.7 × 10^1^	7.5 × 10^1^
**Ideality factor (η)**	**n**	1.32	1.56	1.55	1.55	1.55	1.55	1.59
	**p**	1.17	1.12	1.17	1.17	1.17	1.17	1.39
**Schottky barrier** **height (φb) [eV]**	**n**	0.85	0.81	0.83	0.83	0.83	0.83	0.88
	**p**	1.02	0.99	0.92	0.92	0.92	0.92	1.07

**Table 2:** The electrical parameters of the 600-W MWA and 500-°C RTA NiSi*_x_* SB-TFTs, including the subthreshold swing (*SS*), field-effect mobility (*μ*_*FE*_), threshold voltage (*V_TH_*), on/off current ratio (I_on_ /I_off_), and interface state density (D_it_).

Conduction	Silicidation	Total Parameter				
		*SS* (mV/dec)	Mobility (cm^2^/V∙s)	*V_TH_* (V)	I_on_/I_off_	D_it_ (cm^2^)
**p-type**	**MWA 600 W**	633.4	16.5	2.3	1.1 × 10^7^	9.2 × 10^12^
	**RTA 500 °C**	1201.1	4.9	3.4	7.2 × 10^4^	1.8 × 10^13^
**n-type**	**MWA 600 W**	629.2	20.3	−1.4	6.8 × 10^6^	9.1 × 10^12^
	**RTA 500 °C**	1321.4	4.1	−2.4	5.7 × 10^4^	1.9 × 10^13^

**Table 3:** The charge trapping time (*τ*) extracted from the temperature-dependent ΔV_TH_ of the MWA and RTA NiSi*_x_* SB-TFTs during the PBTS and NBTS tests.

Conduction	Silicidation	PBTS			NBTS		
		25 °C	55 °C	85 °C	25 °C	55 °C	85 °C
**p-type**	**MWA 600 W**	6.0 × 10^5^	1.7 × 10^5^	6.5 × 10^4^	4.1 × 10^5^	1.0 × 10^5^	6.1 × 10^4^
	**RTA 500 °C**	1.2 × 10^5^	3.5 × 10^4^	1.4 × 10^4^	1.6 × 10^5^	4.1 × 10^4^	2.1 × 10^4^
**n-type**	**MWA 600 W**	8.1 × 10^4^	7.3 × 10^4^	1.0 × 10^4^	1.5 × 10^5^	7.0 × 10^5^	3.1 × 10^4^
	**RTA 500 °C**	7.0 × 10^4^	2.4 × 10^4^	8.6 × 10^3^	7.0 × 10^4^	2.5 × 10^4^	1.5 × 10^4^

**Table 4:** The average effective energy barrier height (*E_τ_*) of the MWA- and RTA-NiSi*_x_* SB-TFTs obtained from the PBTS and NBTS tests.

Conduction	Silicidation	Average Effective Energy Barrier [eV]	
		PBTS	NBTS
**p-type**	**MWA 600 W**	0.22	0.19
	**RTA 500 °C**	0.34	0.38
**n-type**	**MWA 600 W**	0.16	0.18
	**RTA 500 °C**	0.32	0.34

## Data Availability

Not applicable.
